# NK Cell–Like Behavior of Vα14i NK T Cells during MCMV Infection

**DOI:** 10.1371/journal.ppat.1000106

**Published:** 2008-07-18

**Authors:** Johnna D. Wesley, Marlowe S. Tessmer, Deanna Chaukos, Laurent Brossay

**Affiliations:** Department of Molecular Microbiology and Immunology and Graduate Program in Pathobiology, Division of Biology and Medicine, Brown University, Providence, Rhode Island, United States of America; Oregon Health Sciences University, United States of America

## Abstract

Immunity to the murine cytomegalovirus (MCMV) is critically dependent on the innate response for initial containment of viral replication, resolution of active infection, and proper induction of the adaptive phase of the anti-viral response. In contrast to NK cells, the Vα14 invariant natural killer T cell response to MCMV has not been examined. We found that Vα14i NK T cells become activated and produce significant levels of IFN-γ, but do not proliferate or produce IL-4 following MCMV infection. In vivo treatment with an anti-CD1d mAb and adoptive transfer of Vα14i NK T cells into MCMV-infected CD1d^−/−^ mice demonstrate that CD1d is dispensable for Vα14i NK T cell activation. In contrast, both IFN-α/β and IL-12 are required for optimal activation. Vα14i NK T cell–derived IFN-γ is partially dependent on IFN-α/β but highly dependent on IL-12. Vα14i NK T cells contribute to the immune response to MCMV and amplify NK cell–derived IFN-γ. Importantly, mortality is increased in CD1d^−/−^ mice in response to high dose MCMV infection when compared to heterozygote littermate controls. Collectively, these findings illustrate the plasticity of Vα14i NK T cells that act as effector T cells during bacterial infection, but have NK cell–like behavior during the innate immune response to MCMV infection.

## Author Summary

An efficient immune response to viral infection requires both innate and adaptive immune cells. Natural killer (NK) cells are a critical innate cellular component of the immune response to murine cytomegalovirus (MCMV). Natural killer T (NK T) cells are non-classical T cells that have the potential to bridge the two arms of the immune system. However, the contribution of NK T cells to the anti-viral immune response has not been extensively studied. In the absence of additional stimuli, NK T cells actively participate in the immune response to MCMV infection. Interestingly, in contrast to their response to bacteria, we demonstrate that only the innate NK T cell arm is activated during viral infection while the adaptive branch, TCR engagement by CD1d, is dispensable. NK T cells display signs of activation in response to viral infection, increased expression of CD25, a rapid decrease in cell number, and production of the anti-viral cytokine IFN-γ. The NK T cell response to MCMV also influences the NK cell activity and the inflammatory cytokine profiles. Understanding the physiological function of these unique T cells in the context of infection will aid in the development of novel therapeutic and preventive treatments for viral infections.

## Introduction

The β-herpes murine cytomegalovirus (MCMV) is a well-characterized model of viral infection that results in a non-replicative, chronic infection of immune-competent animals [1]. MCMV is a cytopathic virus that is known to readily infect peritoneal macrophages, dendritic cells (DC) and hepatocytes, inducing significant pathology in both the spleen and the liver [2]–[5]. The acute response to this virus is dependent on natural killer (NK) cell cytotoxicity and IFN-γ production, as animals deficient in perforin or IFN-γ signaling rapidly succumb to infection [4], [6]–[10].

The hepatic immune environment is greatly influenced by the resident cellular subsets and has been shown to be primarily tolerogenic [11],[12]. The major hepatic lymphocyte population in mice is a distinct family of T cells, Vα14 invariant NK T (Vα14i NK T) cells [13],[14]. Vα14i NK T cells are innate lymphocytes that display an effector memory phenotype, expressing CD69 and CD44 constitutively [15]. They are uniquely capable of rapidly producing T_H_1 and T_H_2 cytokines in response to antigenic stimulation [16]. The Vα14i NK T cell repertoire is highly restricted, characterized by a Vα14-Jα18 rearrangement with an invariant junction preferentially associated with Vβ8.2, Vβ7, or Vβ2 [17],[18]. In response to the ligand α-galactosylceramide (α-GalCer), Vα14i NK T cells interact with and activate other immune cells including NK cells, CD8^+^ T cells, DCs, and macrophages [16]. This immune cell cross-talk is facilitated by direct cell-cell contact and via cytokine release [19]–[22].

Much of the functional significance of Vα14i NK T cell activation in the context of viral infection has been provided by activating the compartment prior to or concomitantly with viral introduction in animal models [23]–[25]. Although this method examines the potential contribution of activated Vα14i NK T cells, it does not examine the physiological function of these T cells in response to viral infection without exogenous stimuli. In the context of other microbial infections, the evidence for direct Vα14i NK T cell involvement is mixed, often being dependent on the type of pathogen [26]–[32].

However, there is indirect evidence that Vα14i NK T cells play a role in anti-viral immune responses. A number of groups have clearly shown that the expression of the antigen-presenting molecule CD1d is often down-regulated by viruses in a myriad of ways, including protein degradation, alterations in transcription, or endosomal sequestration [33]–[35]. Vα14i NK T cells have also been shown to be preferential targets of infection and virus-induced cell death [36],[37]. This indicates that Vα14i NK T cells may have a potential role in the anti-viral response and it is advantageous for the pathogen to prevent their activation.

To directly assess the role of Vα14i NK T cells in the innate anti-viral response, their activation status was examined following MCMV infection in vivo. We found that Vα14i NK T cells up-regulate the high affinity IL-2 receptor-α, CD25, produce IFN-γ, but do not undergo proliferation. Importantly, we demonstrate that CD1d is dispensable for Vα14i NK T cell activation and cytokine release in the context of MCMV. However, IFN-α/β and IL-12 are both partially required for optimal activation of the Vα14i NK T cells in response to infection. We also show that in the absence of α-GalCer treatment, Vα14i NK T cells contribute significantly to the overall cytokine response and amplify NK cell-derived IFN-γ production. Collectively, our findings demonstrate a role for the NK T cells in innate sensing of viral pathogens in an unanticipated NK cell-like manner.

## Materials and Methods

### Mice

Inbred C57BL/6 and B6.SJL-Ptprca/BoAiTac mice were purchased from Taconic Laboratory (Hudson, NY). B6.IL-12p40^−/−^ mice were purchased from the Jackson Laboratory (Bar Harbor, ME). B6.CD1d^−/−^ mice (a generous gift from Dr. L. Van Kaer, Vanderbilt University, Nashville, TN) and B6.Jα18^−/−^ mice (kindly provided by Dr. M. Taniguchi, Riken Research Center for Allergy and Immunology, Yokohoma, Japan) were bred, crossed to the B6 (>10 generations) to generate wild-type, heterozygous, and knock-out littermates. Female IFN-α/βR1^−/−^ mice originally generated on the 129.SvEv background and backcrossed on to the C57BL/6 background were kindly provided by Dr. M. Aguet [38] and bred in our facility. All mice, except B6 mice, were bred in pathogen-free breeding facilities at Brown University (Providence, RI). All experiments were conducted in accordance with institutional guidelines for animal care.

### Virus & Infection Protocols

Stocks of Smith strain MCMV salivary gland extracts were prepared as previously described [39]. Infections were initiated on day 0 with 5×10^4^ plaque-forming units (PFU), administered via i.p. injection. For survival studies, 3×10^5^ PFU were administered via i.p. injection. For antibody-blocking experiments, mice received blocking CD1d mAb (0.3 mg; clone 1B1; BD Pharmingen) or rat IgG control Abs in PBS at the time of the infection.

### Lymphocyte Isolation

To obtain splenic lymphocytes, spleens were minced, passed through nylon mesh (Tetko, Kansas City, MO), washed once in 2% PBS-serum and cell suspensions were layered on Lympholyte-M (Cedarlane Laboratories Ltd., Canada). Hepatic lymphocytes were prepared by mincing and passage through a 70 mm nylon cell strainer (Falcon, Franklin Lakes, NJ). After washing 3 times in 2% PBS-serum, cell suspensions were layered on a two-step discontinuous Percoll gradient (Pharmacia Fine Chemicals, Piscataway, NJ). Splenocytes and hepatic lymphocytes were collected after centrifugation for 20 min at 900×g.

### Antibodies and Reagents

CD19-FITC, TCRβ-FITC, CD11b-FITC, CD11c-FITC, NK1.1-PE, CD1d-PE, B220-PerCP-Cy5, KLRG1-allophycocyanin, CD25-APC, and TCRβ-allophycocyanin were all purchased from eBioScience (San Diego, CA). NK1.1-PerCp-Cy5.5, CD11b-PerCp, CD4-PerCp, CD8-PerCp, CD11c-allophycocyanin, B220-allophycocyanin, IFN-γ-allophycocyanin and isotype control were purchased from BD Pharmingen (San Diego, CA). For NK T cell identification, CD1d tetramers were obtained from the National Institute of Allergy and Infectious Disease MHC Tetramer Core Facility at Emory University (Atlanta, GA). Additionally, the following mAbs were purchased from BD Pharmingen and used for ELISA: IFN-γ mAbs (clone R4-6A2, and clone XMG1.2), IL-4 mAbs (clone 4B11 and BVD6-24G2), IL-2 mAbs (purified JES6-N37-1A12 and biotinylated JES6-5H4) and streptavidin-peroxidase.

### Adoptive Transfer of Enriched NK T Cells

Hepatic lymphocytes were isolated as described above from congenic C57BL/6.SJL mice. For enrichment of hepatic NK T cells, cells were first depleted of CD8^+^, CD11c^+^, CD11b^+^, and CD19^+^ cells using the AutoMACS (Miltenyi Biotec) as instructed by the manufacturer. 5–8×10^6^ cells were transferred via tail vein injection into Jα18^−/−^ or CD1d^−/−^ mice. For NK T cell positive selection hepatic lymphocytes were stained with anti-NK1.1 and anti-CD5 mAbs or with anti-NK1.1 and CD1d tetramer and sorted using a FACSAria (BD Biosciences). At the time of transfer (1×10^6^ cells per mouse), mice were infected with 5×10^4^ pfu MCMV. At 1.5 days post-infection, animals were sacrificed and the donor population analyzed for IFN-γ production.

### Serum Cytokine Measurement

For all serum-based measurements, blood was collected via cardiac puncture. Serum was separated from the cellular fraction by centrifugation at 14,000 rpm at 4°C for 30 minutes. Serum levels of cytokines were measured by ELISA or using the cytometric bead array (CBA) mouse inflammation kit (BD Pharmingen).

### Flow Cytometric Analysis

Following lymphocyte isolation, cells were suspended in PBS containing 2% FCS. Cells were then incubated with 2.4G2 anti-Fc receptor mAb and stained with indicated antibodies. Cells were then fixed in 2% paraformaldehyde in PBS. Intracellular staining for IFN-γ protein was performed using the Cytofix/Cytoperm kit (BD PharMingen). Depending on the experiment and the tissue, 2.5×10^5^–1×10^6^, events were collected on a FACSCalibur or FACSAria. The data were analyzed using CellQuest software or Diva software (Becton Dickinson, Franklin Lakes, NJ).

### Statistical Analysis

Statistical significance, designated as a p-value ≤0.05, was determined by paired, 2-tailed Student's T-test.

## Results

### Vα14i NK T cells are activated but do not proliferate in response to MCMV infection in vivo

**Figure 1:**
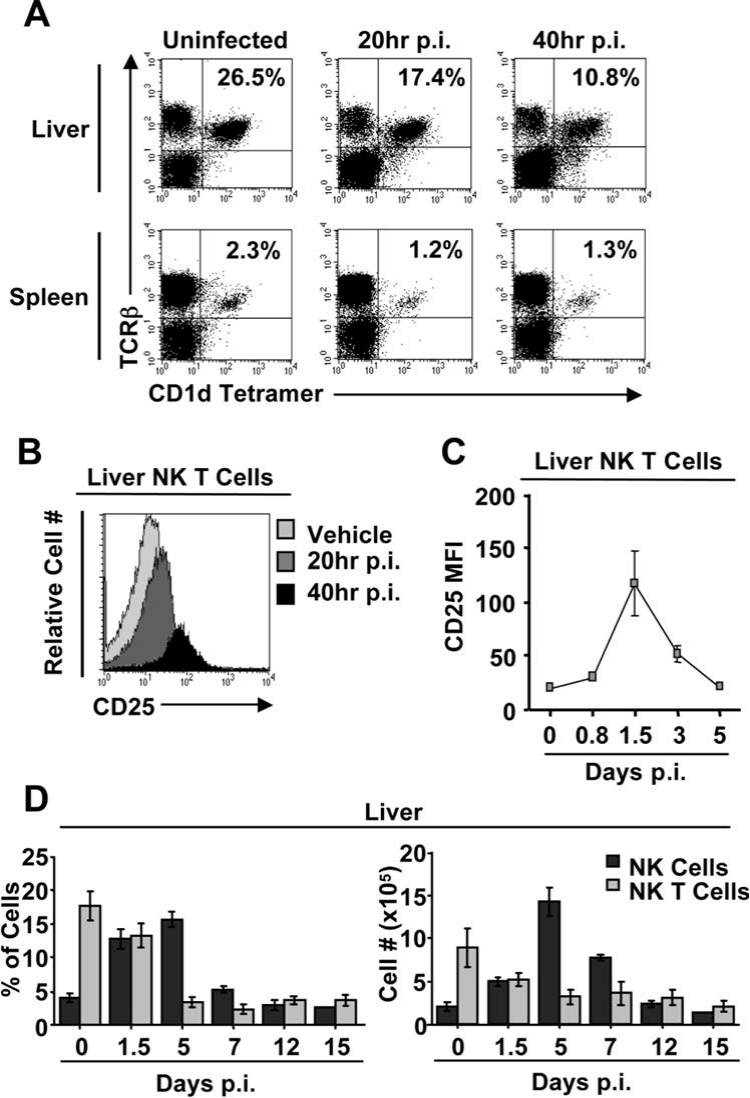
Vα14i NK T cells are activated but do not expand in response to MCMV infection in vivo. *A*, Splenic and hepatic leukocytes were isolated from MCMV infected or vehicle treated mice at 20 and 40 hrs post-infection and the Vα14i NK T cell compartment was analyzed by staining with TCR-β and α-GalCer-loaded CD1d tetramer. *B*, Hepatic Vα14i NK T cells were analyzed for the surface expression level of CD25 at 20 and 40 hrs p.i. compared to vehicle treated mice. *C,* The average expression level of CD25 on the surface of Vα14i NK T cells, average MFI±SD is shown. *D,* Hepatic leukocytes were isolated from MCMV infected or vehicle treated mice at the indicated days post-infection. The Vα14i NK T cell compartment was analyzed by staining with TCR-β and α-GalCer-loaded CD1d tetramer and the NK cell compartment was analyzed by gating on the NK1.1^+^TCRβ^−^ cells. The percentage and absolute number of Vα14i NK T and NK cells is shown. Results are representative of 3 to 5 independent experiments.

It is well documented that NK cells are necessary for the innate anti-viral immune response to MCMV infection [6],[40]. However, it is unclear if naïve Vα14i NK T cells also participate in this innate immune response. To address this issue, wild type B6 mice were infected for 20–40 hrs and the activation status of Vα14i NK T cells was examined in the liver and spleen. In both tissues, Vα14i NK T cells display signs of activation, a decrease in NK T cell numbers (Fig. 1A), and CD25 up-regulation by 20 hours post-infection (Fig. 1B and data not shown).

CD1d dependent Ag recognition by Vα14i NK T cells induces their expansion [41]. Additionally, Vα14i NK T cells have been shown to proliferate in response to infection with LPS negative bacteria [31],[42]. However, in the context of MCMV infection, the Vα14i NK T cell compartment does not expand in either number or frequency (Fig. 1D), even at the peak of activation as assessed by CD25 expression (Fig. 1C). We also performed an intra-cellular staining for TCR at different days post MCMV infection. We found that most of the cells were double positive for intracellular and cell surface TCR, ruling out a possible lack of detection of the Vα14i NK T cells due to TCR internalization (data not shown). In contrast to Vα14i NK T cells, NK cells expand during MCMV infection (Fig. 1D). Furthermore, the percentage of CD25^+^ Vα14i NK T cells rapidly declines in comparison to the protracted decrease in the percent of NK cells positive for the terminal maturation marker, KLRG1 (data not shown).

### Vα14i NK T cells produce IFN-γ in response to MCMV infection

**Figure 2:**
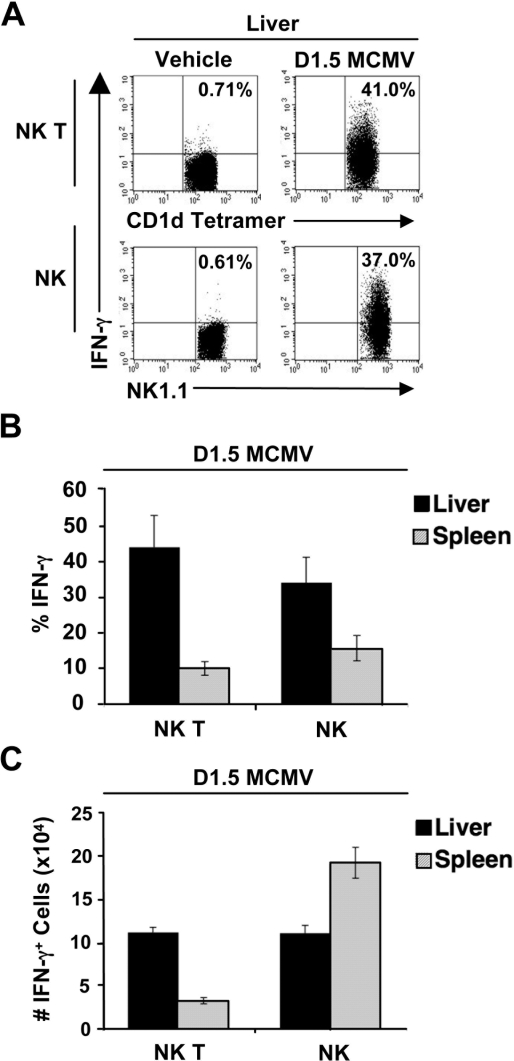
Vα14i NK T cells produce IFN-γ in response to MCMV infection. *A*, Following surface staining, hepatic Vα14i NK T cells and NK cells were fixed, permeablized and stained for intracellular IFN-γ. *B,* The frequency and *C,* number of IFN-γ^+^ Vα14i NK T and IFN-γ^+^ NK cells, average±SD, is shown for infected B6 at day 1.5 post-infection. Results shown are representative of 2 to 5 independent experiments.

Vα14i NK T cells produce IFN-γ as early as 30 hours post-infection (data not shown), peaking at day 1.5 post-infection (Fig. 2A). At this time point, the frequency of IFN-γ^+^ Vα14i NK T cells is comparable to the frequency of IFN-γ^+^ NK cells in both spleen and liver (Fig. 2B). In the spleen, despite a similar frequency, the number of IFN-γ^+^ Vα14i NK T cells is lower than the number of IFN-γ^+^ NK cells. However, the number of IFN-γ^+^ Vα14i NK T cells is similar to the number of IFN-γ^+^ NK cell in the liver at day 1.5 post-infection (Fig. 2C). This indicates that these two subsets of cells contribute equally to the overall amount of IFN-γ in the liver. Notably, Vα14i NK T cells do not produce detectable amounts of IL-4 during MCMV infection in either tissue (data not shown).

### CD1d is dispensable for Vα14i NK T cell activation in response to MCMV infection

**Figure 3:**
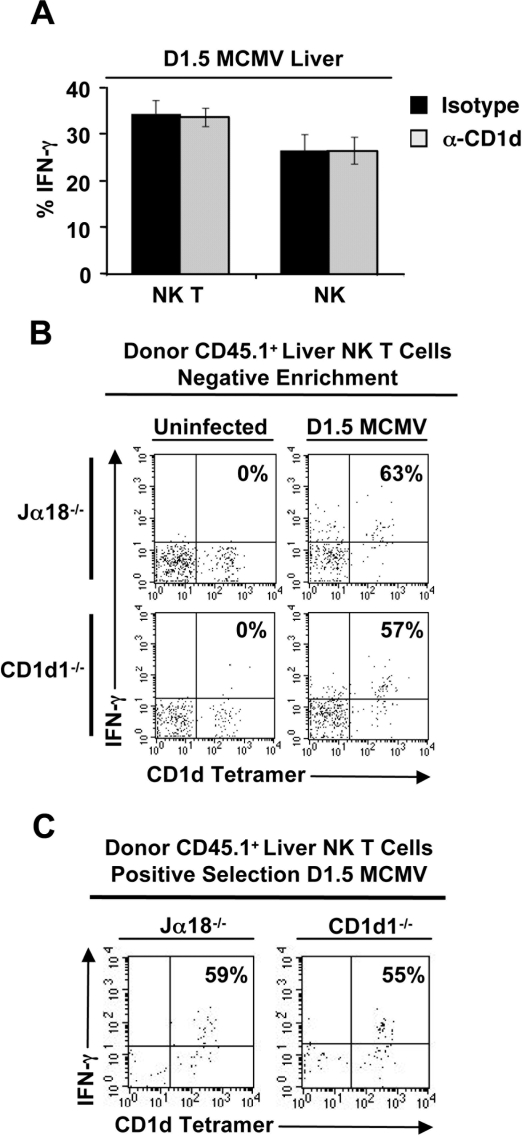
CD1d is dispensable for Vα14i NK T cell activation in response to MCMV. *A*, B6 mice were injected with 300 µg of anti-CD1d mAb or isotype control and infected with 5×10^4^ pfu/mouse MCMV or vehicle control. Hepatic and splenic lymphocytes were isolated from the host animals at day 1.5 post-infection and examined for the percentage of IFN-γ^+^ Vα14i NK T cells and IFN-γ^+^ NK cells. IFN-γ was not detectable in vehicle treated animals (data not shown). The results are representative of 2 separate experiments. *B*, Hepatic leukocytes were isolated from wild type, congenic B6 mice and depleted of CD8^+^, CD11b^+^, CD19^+^, and CD11c^+^ cells to enrich for Vα14i NK T cells prior to injection via tail vein into CD1d^−/−^ or Jα18^−/−^ mice infected with 5×10^4^ pfu/mouse MCMV or vehicle control. Hepatic and splenic lymphocytes were isolated from the host animals at day 1.5 post-infection and examined for the percentage of IFN-γ^+^ Vα14i NK T cells. The results are representative of 4 separate experiments. *C*, Hepatic leukocytes were isolated from wild type, congenic B6 mice and CD5^+^NK1.1^+^ cells were sorted prior to injection via tail vein into CD1d^−/−^ or Jα18^−/−^ mice infected with 5×10^4^ pfu/mouse MCMV or vehicle control. Hepatic and splenic lymphocytes were isolated from the host animals at day 1.5 post-infection and examined for the percentage of IFN-γ^+^ Vα14i NK T cells. The results are representative of 3 independent experiments.

In order to investigate whether MCMV induced activation of Vα14i NK T cells requires CD1d, B6 mice were treated with a blocking CD1d mAb or control antibody and infected with MCMV. On day 1.5 post-infection, the percentage of hepatic IFN-γ^+^ Vα14i NK T cells in mice that received the anti-CD1d mAb or control IgG was comparable (Fig. 3A). Similar results were observed in the spleen (data not shown). To directly assess the contribution of CD1d-mediated Ag presentation to MCMV-induced activation and cytokine production from Vα14i NK T cells, adoptive transfer experiments were performed. Negatively selected (purity >70%) or positively selected hepatic Vα14i NK T cells (purity >95%) from congenic wild-type B6.SJL mice were adoptively transferred into CD1d^−/−^ or Jα18^−/−^ deficient hosts. The recipient mice were simultaneously infected with MCMV for 1.5 days and the percentage of IFN-γ^+^ Vα14*i* NK T cells was determined. Regardless of the host expression of CD1d, donor Vα14*i* NK T cells produced similar amounts of IFN-γ following MCMV infection in vivo (Fig. 3B & 3C). Taken together, the results indicate that CD1d is dispensable during MCMV induced activation of Vα14i NK T cells.

### Vα14i NK T cell IFN-γ production in response to MCMV infection is partially dependent on IFN-α/β and IL-12

**Figure 4:**
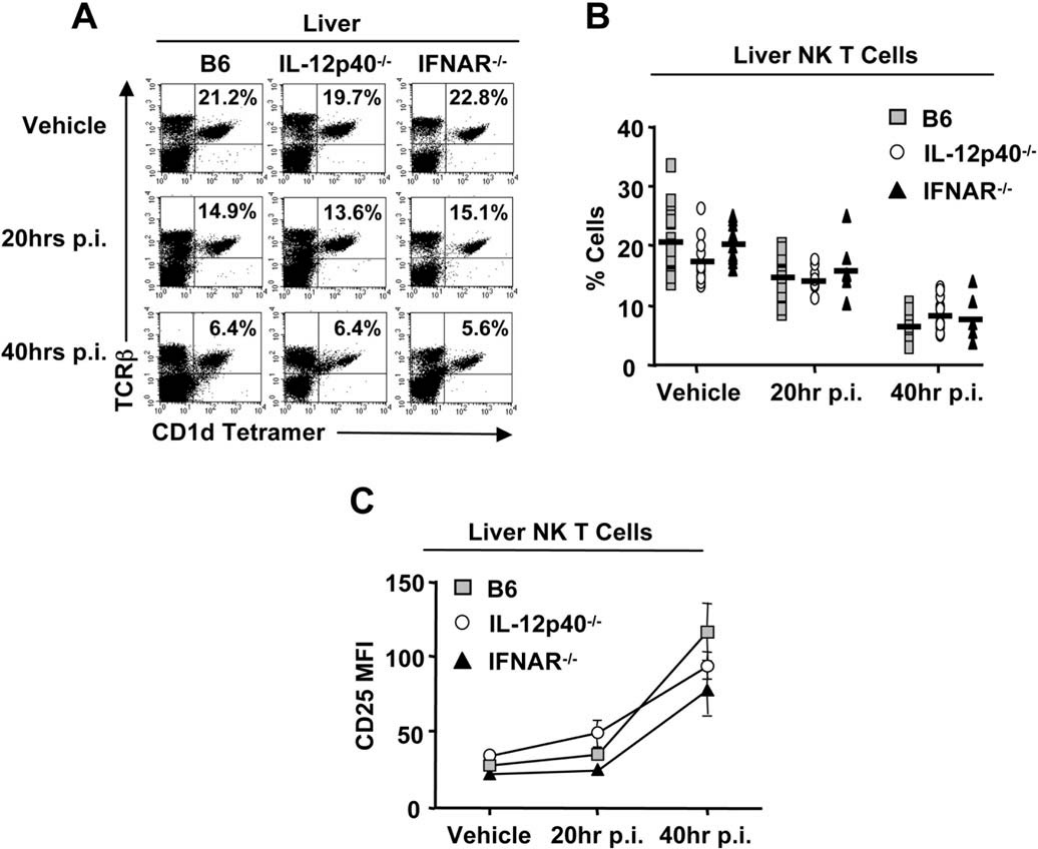
Vα14i NK T cell activation in response to MCMV is independent of IFN-αβ and IL-12. *A and B*, Hepatic leukocytes were isolated from MCMV infected or vehicle treated B6, IL-12p40^−/−^, and IFNα/βR1^−/−^ mice at 20 and 40hrs post-infection and the Vα14i NK T cell compartment was analyzed by staining with TCR-β and α-GalCer-loaded CD1d tetramer. The percentage of Vα14i NK T cells in the liver of B6, IL-12p40^−/−^, and IFNα/βR1^−/−^ mice, uninfected and at indicated time points post-infection is shown in *B* as average±SD. *C*, Hepatic Vα14i NK T cells were analyzed for the surface expression of CD25 at 20 and 40 hrs p.i. compared to vehicle treated B6, IL-12p40^−/−^, and IFNα/βR1^−/−^ mice. The MFI of CD25 expression on the Vα14i NK T cells is shown as average±SD. Results shown are representative of 2 to 5 independent experiments.

**Figure 5:**
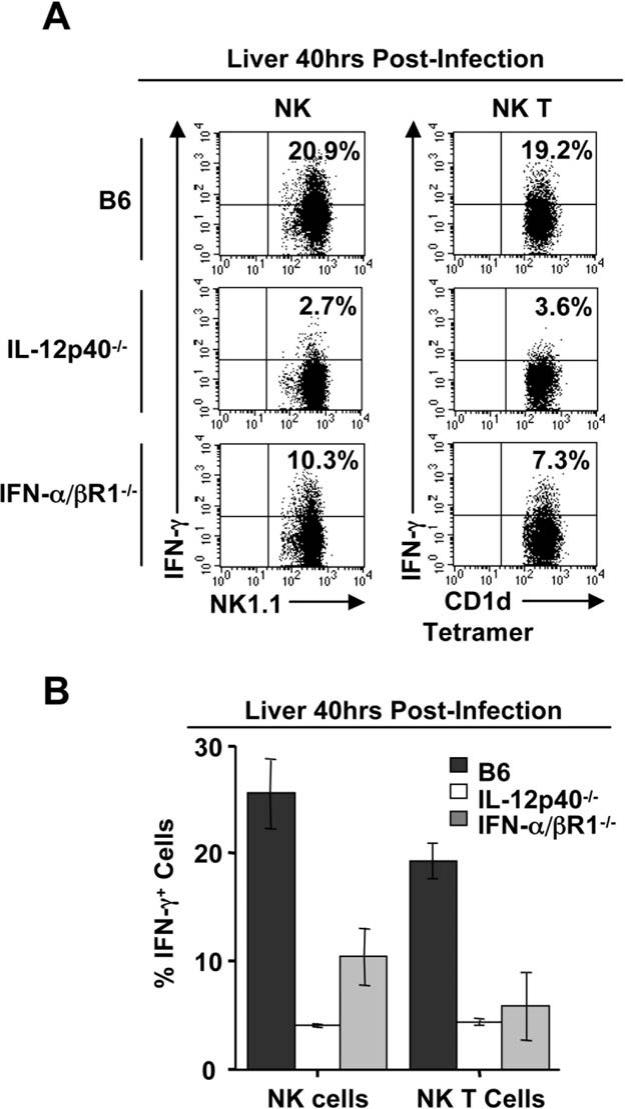
Optimal Vα14i NK T cell IFN-γ response requires IFN-α/β and IL-12. *A,* Hepatic leukocytes isolated from B6, IL-12p40^−/−^, and IFNα/βR1^−/−^ mice at 40 hrs p.i. were stained with α-GalCer CD1d tetramer, TCRβ, and NK1.1 followed by permeabilization and stained for intracellular IFN-γ and compared to vehicle treated mice. IFN-γ^+^ Vα14i NK T cells and NK cells are shown. *B*, The percent of IFN-γ^+^ Vα14i NK T cells and NK cells, average±SD, is shown for infected B6, IL-12p40^−/−^, and IFNα/βR1^−/−^ mice. IFN-γ was not detectable in vehicle treated animals (data not shown). Results shown are representative of 2 to 5 independent experiments.

High levels of IFN-α/β and bioactive IL-12 characterize the innate immune response to MCMV infection in vivo [43]. In the absence of either cytokine, the innate anti-viral response is fatally impaired [39],[44]. Here, MCMV infection of IFN-α/βR1^−/−^ and IL-12p40^−/−^ mice further reveals that the activation of Vα14i NK T cells at both 20 and 40 hours post-infection is independent of IL-12 and IFN-α/β, as assessed by the percentage of NK T cells (Fig. 4A & 4B) and CD25 expression (Fig. 4C). However, Vα14i NK T cell-derived IFN-γ is highly dependent on IL-12 in the liver (Fig. 5) and spleen (data not shown), similar to NK cells (Fig. 5). Notably, the Vα14i NK T cell-derived IFN-γ response is reduced by ∼50% in MCMV infected IFN-α/βR1^−/−^ mice, further mimicking NK cell dynamics (Fig. 5).

### Vα14i NK T cells amplify NK cell IFN-γ production and inflammatory cytokine production during MCMV infection

**Figure 6:**
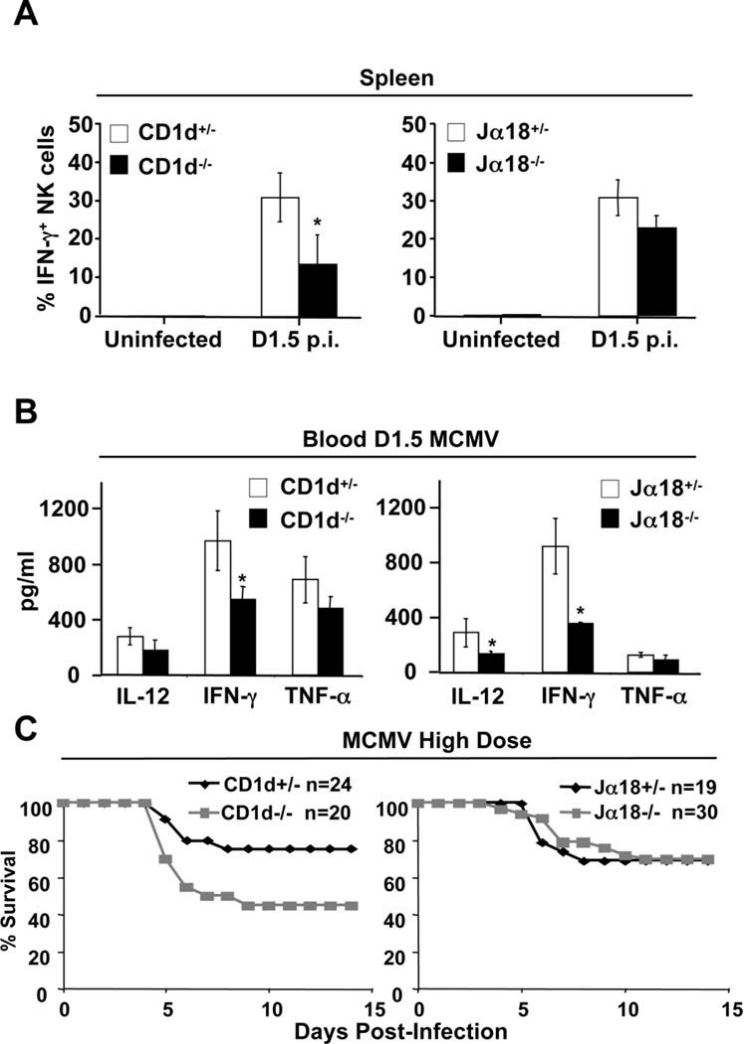
The innate immune cytokine response is impaired in the absence of Vα14i NK T cells. *A*, Splenic leukocytes were isolated from uninfected or MCMV infected CD1d^−/−^ and CD1d^+/−^ littermates or Jα18^−/−^ and Jα18^+/−^ littermates at 1.5 days post-infection and analyzed for intracellular IFN-γ. The percentage of IFN-γ^+^ NK cells is shown. The results are representative of 5 independent experiments (*, P<0.05). *B*, Serum levels of cytokines were measured by ELISA or using the cytometric bead array inflammation kit at 1.5 days post-infection (*, P<0.05). The results are representative of 3 independent experiments. *C*, Percent survival following high dose MCMV infection.

Activated Vα14i NK T cells interact with and activate other immune cells such as NK cells, which subsequently produce cytokines [19],[20],[45]. To investigate the downstream consequences of Vα14i NK T cell absence, we measured both NK cell-derived IFN-γ and serum inflammatory cytokines in infected NK T cell deficient mice. The activation of NK cells in the spleen, as determined by IFN-γ production, was significantly reduced in CD1d^−/−^ mice when compared to heterozygous littermates (Fig. 6A). Similarly, although not significant, a reproducible reduction of NK cell IFN-γ was also observed in MCMV infected Jα18^−/−^ mice compared to Jα18^−/+^ littermates in five independent experiments (Fig. 6A). Interestingly, an overall reduction of the inflammatory cytokine profile (IL-12, IFN-γ and TNF-α) was seen in the blood of Jα18^−/−^ animals in comparison to heterozygous littermate controls at 1.5 days post-MCMV infection in vivo (Fig. 6B). Likewise, inflammatory cytokines were diminished in CD1d^−/−^ mice compared to CD1d^+/−^ littermates. However, in the latter case, while IL-12 and TNF-α were reproducibly decreased, only IFN-γ was reduced significantly. Notably, IL-4 and IL-10 were not detectable in the serum of infected animals.

### CD1d^−/−^ mice but not Jα18^−/−^ mice are more susceptible to high dose MCMV infection than their heterozygous littermates

To address whether Vα14i NK T cells and/or CD1d participate in the early control of MCMV infection, CD1d^−/−^ and Jα18^−/−^ animals, as well as littermate controls were used in survival studies with high dose MCMV infection. CD1d^−/−^ mice were more susceptible than CD1d^+/−^ mice, as only 50% of the CD1d^−/−^ mice lived beyond day 15. While Jα18^−/−^ animals were not more susceptible than their littermate, heterozygous controls (Fig. 6), they were significantly more susceptible than wild-type B6 mice from outside vendors (not shown). Taken together, these results demonstrate that Vα14i NK T cells influence NK cell activity, the inflammatory cytokine profiles, and that both Vα14i NK T cells and other CD1d restricted T cells are necessary for an optimal immune response to MCMV.

## Discussion

The function of NK cells in anti-viral immunity has been documented; however, evidence for a direct Vα14i NK T cell role has not been examined extensively. The results presented in this report show that Vα14i NK T cells sense MCMV infection in vivo, without exogenous stimuli, such as α-GalCer. However, in contrast to bacterial infection, we provide evidence that MCMV induced Vα14i NK T cell activation is TCR independent.

Vα14i NK T cells can be activated directly by agonist glycolipids presented by CD1d. For instance, α-GalCer immunization of B6 mice leads to IL-12 independent activation of Vα14i NK T cells [16]. In this case, Vα14i NK T cells release copious amount of IL-4 and IFN-γ and subsequently proliferate. Gram-negative LPS-negative α-proteobacteria, such as *Sphingomonas*, *Ehrlichia*, *Rickettsia*, and *Borrelia*, express such agonist lipids and can directly activate Vα14i NK T cells [31],[32],[42],[46]. Bacteria that do not express agonist glycolipids have been reported to activate Vα14i NK T cells through up-regulation of self glycolipids and/or IL-12 production through recognition of endogenous lysosomal glycosphingolipids, such as iGb3, presented by LPS-activated dendritic cells [31],[47]. Therefore, there are two major mechanisms for the activation of Vα14i NK T cells against bacteria either via cognate Ag or via self-Ag with APC derived cytokines [48]. Using MCMV infection in vivo, we now demonstrate a novel activation pathway for Vα14i NK T cells, mediated principally by inflammatory cytokines.

MCMV induced activation of Vα14i NK T cells clearly differs from the two mechanisms described in response to bacterial infection. First, as opposed to α-GalCer administration [41],[49] and α-proteobacteria infection [31], Vα14i NK T cells do not proliferate nor produce IL-4 following MCMV-induced activation. Second, while IL-12 is not required for optimal stimulation of Vα14i NK T cells in response to α-GalCer or *sphingomonas*-derived glycolipids [20],[42], here we show that Vα14i NK T cell cytokine production is impaired in IL-12 deficient animals in response to MCMV infection. Finally, in vivo CD1d blocking experiments and adoptive transfer of Vα14i NK T cells into CD1d^−/−^ mice demonstrates that CD1d is dispensable for MCMV induced activation of these lymphocytes. It should be noted that LPS-induced Vα14i NK T cell-derived IFN-γ in vitro does not require CD1d-mediated Ag presentation, instead exposure to IL-12 and IL-18 is sufficient to activate these cells [50].

These data raise the question of why do bacteria such as *Salmonella typhimurium* and *Staphylococcus aureus* activate Vα14i NK T cells in both an IL-12 and CD1d dependent manner, while MCMV induced activation of Vα14i NK T cells is CD1d independent? There are several non-mutually exclusive possibilities that could explain this apparent discrepancy. First, viruses unlike bacteria, do not encode enzymatic machinery for lipid synthesis. Second, the peak of the cytokine response to MCMV occurs relatively early when compared to bacteria, allowing for possible Vα14i NK T cell activation to occur prior to cytokine-driven self-Ag activation. Third, while Gram negative bacteria cytokine-driven self-Ag activation of Vα14i NK T cells was demonstrated in vitro using bone marrow derived DCs [31],[47], it has been recently demonstrated that plasmacytoid dendritic cells (pDCs) are the quasi-exclusive source of IFN-α/β, IL-12 and TNF-α early during MCMV infection [51]. It is therefore possible that depending on the pathogen and the source and/or phenotype of recruited CD1d^+^ DCs may lead to differential activation of Vα14i NK T cells.

pDCs and dendritic cells recognize MCMV through TLR9, an essential component of the innate immune defense against MCMV. Tabeta *et al* have shown that the Vα14i NK T cell response to MCMV is impaired in TLR9^−/−^ mice [52]. Interestingly, the serum level of both IFN-α and IL-12 is reduced in TLR9^−/−^ mice following MCMV infection [52],[53], supporting our findings that the absence of these cytokines impairs the Vα14i NK T cell response to MCMV. However, activation of Vα14i NK T cells by TLR9-stimulated dendritic cells was recently shown to be CD1d dependent [54]. The latter study was performed in vitro using BMDCs grown for 14 days prior to being pulsed with CpG for 16 hours. This procedure clearly differs from MCMV infection in vivo where the peak of the Vα14i NK T cell response is at 1.5 days post-infection. It is possible that some pathogen-derived products such as CpG may increase endogenous glycolipid presentation during the anti-viral response but that this process is not yet initiated at the peak of the innate response to MCMV.

In the context of MCMV infection, we failed to detect an expansion of the Vα14i NK T cells. Instead, there is a gradual loss of these cells in the liver and spleen following infection. Presumably, the lack of TCR engagement by CD1d during MCMV infection promotes the activation of Vα14i NK T cells that release cytokines but do not proliferate and subsequently die. It is also possible that Vα14i NK T cells preferentially undergo virus-induced apoptosis similarly to what has been reported during the anti-viral response against lymphocytic choriomeningitis virus infection in vivo [36].

The early immune response to MCMV is characterized by the production of high levels of inflammatory cytokines [51],[55]. Type I IFNs, which are critical for anti-viral immunity, can be detected very early following MCMV infection and mediate the proliferation and survival of activated lymphocytes [56]. Additionally, the classical T_H_1-promoting cytokine, IL-12, is also produced early and is necessary for NK cell-derived IFN-γ [57]. Infection of mice deficient in either IFN-α/β signaling or bioactive IL-12 clearly demonstrates that neither cytokine alone is sufficient to mediate an optimal IFN-γ response from Vα14i NK T cells in response to MCMV. IFN-α/β is thought to negatively regulate the production of IFN-γ via inhibition of IL-12 thus ensuring that IFN-γ does not prematurely inhibit proliferation [58]. The timing of IL-12 production may be critical for Vα14i NK T cells as MCMV infected IFN-α/βR1^−/−^ mice produce high levels of IL-12 [56], yet we show that Vα14i NK T cell-derived IFN-γ is impaired in IFN-α/βR1^−/−^ mice. It is also currently unclear if IFN-α/β acts directly on this innate T cell population in the context of MCMV infection. These issues warrant further inquiry.

Vα14i NK T cells are widely appreciated for their rapid cytokine production and ability to interact with and activate both innate and adaptive immune cells [15]. The cross-talk between Vα14i NK T cells and NK cells in the context of α-GalCer-mediated stimulation requires IFN-γ and IL-12 production to promote optimal NK cell activation [19],[20]. Vα14i NK T cell-mediated activation of NK cells has been shown to be required for anti-tumor immunity [59],[60] but not for viral infection. We show that in the absence of the Vα14i NK T cell population, the NK cell response to MCMV in vivo is impaired. Notably, Vα14i NK T cells also mediate activation and maturation of DCs and macrophages [61],[62], cells critical for the induction of the anti-viral immune response via production of high levels of key inflammatory cytokines such as IFN-α/β and IL-12 [62]. We are currently investigating the possibility that the function of these subsets in response to MCMV infection may also be altered in the absence of Vα14i NK T cells.

Our data suggest that Vα14i NK T cells contribute to the overall immune response to MCMV. However, it has been shown that at a low infecting dose, Jα18^−/−^ mice and B6 wild-type control animals have equivalent viral titers [25]. Using Vα14i NK T cell deficient mice and littermate controls, we examined the pathological impact of Vα14i NK T cell absence during high dose MCMV challenge. While both Jα18^−/−^ and CD1d^−/−^ animals are more susceptible than wild-type B6 mice from outside vendors, only CD1d^−/−^ mice are less resistant than heterozygous littermate control mice to high dose MCMV infection. This suggests that Vα14i NK T cells as well as other CD1d restricted T cells are required for an optimal immune response to MCMV.

Collectively, the findings presented in this report indicate that Vα14i NK T cells actively participate in the innate immune response to MCMV in vivo and directly impact the quality of the immune response. However, the mechanism of their activation differs from bacterial induced activation and in this case, NK T cell functions mirror NK cell functions. These data define a previously unappreciated role for CD1d restricted T cells in anti-viral immunity and provide additional insight into the affect of innate immune manipulation on the overall outcome of an immune response.
